# Reduced miR-126 expression facilitates angiogenesis of gastric cancer through its regulation on VEGF-A

**DOI:** 10.18632/oncotarget.2662

**Published:** 2014-11-15

**Authors:** Hongxia Chen, Lingmin Li, Shaojun Wang, Yupeng Lei, Qi Ge, Nonghua Lv, Xiaodong Zhou, Changyan Chen

**Affiliations:** ^1^ Department of Gynaecology and Obstetrics, The First Affiliated Hospital of Nanchang University, Nanchang, China; ^2^ Department of Gastroenterology, General Hospital of Jinan Military Command, Jinan, China; ^3^ Department of Ophthalmology, Affiliated Hospital of Academy of Military Medical Sciences, Beijing, China; ^4^ Department of Gastroenterology, The First Affiliated Hospital of Nanchang University, Nanchang, China; ^5^ Center for Drug Discovery, Northeastern University, Boston, USA

**Keywords:** miR-126, gastric cancer, angiogenesis, VEGF, Akt/m-TOR phosphorylation

## Abstract

miR-126 is an endothelial-specific microRNA essential for governing vascular integrity and angiogenesis. Its role in tumor angiogenesis of gastric cancer (GC) is unclear. This study aimed at determining the role of miR-126 in GC angiogenesis. Down-regulation of miR-126 was found to inversely correlate with an increased microvessel density (MVD) and vascular endothelial growth factor A (VEGF-A) expression in gastric cancer tissues. Bioinformatics analysis and luciferase reporter assay revealed that miR-126 directly targeted the 3′-untranslated region (3′-UTR) of VEGF-A mRNA. In addition, the restoration of miR-126 expression by lentivirus-miR-126 (Lenti-miR-126) transfection obviously reduced the expression of VEGF-A and the activition of its downstream genes, Akt, mTOR and Erk1/2 in gastric cancer cell lines SGC-7901, MKN-28 and MKN-45. In contrast, the down-regulation of miR-126 expression by lentivirus-anti-miR-126 (Lenti-anti-miR-126) transfection obviously up-regulated the expression of VEGF-A and its downstream signaling pathways. *In vivo* xenograft mice model experiments clarified the down-regulation of VEGF-A and MVD as well as inhibition of tumor growth by up-regulation of miR-126. Overall, the results from our study suggested that miR-126 could suppress tumor growth and tumor angiogenesis of GC through VEGF-A signaling, and it is a novel potential therapeutic target for GC.

## INTRODUCTION

Gastric cancer has long been one of the world's major cancer especially in Asian countries. Although the overall survival rate for patients with gastric carcinoma has increased, as a result of improved detection of early cancer and wider implementation of radical surgery, it still ranked the fourth most common cancer and might be the second leading cause of cancer death world widely [1–3]. Gastric cancer is aggressive in essence and a large number of even those patients who have early stage disease will eventually die from recurrence after definitive therapy. Patients with advanced stomach cancer have very limited options for target agents and conventional chemotherapy has remained the standard of treatment. As a result, the overall outcome of patients is barely satisfactory due to metastasis and recurrence [1, 4]. Since angiogenesis is desirable for the development, progression, and metastasis of various cancers [5], it has been wildly studied and antiangiogenesis continues to be the repeating theme of cancer therapy of the modern era. Although successful in other tumor types, the data from highly anticipated antiangiogenic agents from both the small molecule tyrosine kinase inhibitors and monoclonal antibodies for gastric cancer had been frustrating [6]. There is an urgent call for finding new therapeutic targets for anti-angiogenesis of gastric cancer.

Angiogenesis, the sprouting of new blood vessels from pre-existing ones, is essential to the body's development as well as invasive tumor growth and tumor pathogenesis. During the past few decades, many studies have revealed that angiogenesis is activated by various signaling molecules and growth factors, including transforming growth factor beta (TGF-β), fibroblast growth factor (FGF) and vascular endothelial growth factor (VEGF). It is well documented that VEGF plays a pivotal role in modulating endothelial cell function, such as blood vessel formation during embryonic development, and plays a vital role in the proliferation, migration, and invasion of vascular endothelial cells. During development of tumors, the tumor cells suffered from hypoxia, and secrete angiogenic factors such as VEGF, which activate dormant endothelial cells to form new capillary [5].

microRNAs (miRNAs), which are endogenous 21–23 nucleotide (nt) non-coding RNAs, play important roles in tuning normal cell activity [7]. Malfunction of miRNAs can leads to tumorigenesis [8–11]. Through microarray analysis of miRNAs in various tumors and normal control tissues, specific miRNA expression profiles can be characterized for certain types of tumors [12–18]. Many microRNAs have been found to involve in the physiological and pathological processes of angiogenesis [19, 20]. miRNA-126 (miR-126), which is identified in the endothelial cell of blood vessels, controls angiogenesis by binding to several transcripts [21–23]. In addition, the results from many studies have shown that miR-126 is either a tumor suppressor or an oncogene depending on the type of cancer. Over-expression of miR-126 was found in acute myeloid leukemia [24] and miR-126 expression reduced in colorectal cancer [25, 26], prostate cancer [27], breast cancer [28], oral cancer [29], lung cancer [30] and gastric cancer [14]. Noticeably, the reduction of miR-126 was related to the increase of capillary density in lung cancer tissue, and restoration of miR-126 in lung cancer obviously reduced the VEGF expression level and micro-vessel density (MVD), thus could inhibit the growth of lung cancer [30, 31].

However in gastric cancer, the effect of miR-126 on angiogenesis remains unclear. In the present study, the effects of miR-126 on gastric cancer angiogenesis as well as its relative molecular mechanism were investigated using *in situ* human gastric cancer tissues, *in vitro* gastric cancer cell lines and *in vivo* mouse model systems. We identified the down-regulated expression of miR-126 in gastric cancer tissue compared to the normal gastric mucosa as well as an enhanced expression of VEGF-A and its downstream signaling molecules in several gastric cancer cell lines. In addition, our presented evidences indicated that miR-126 binded directly to the VEGF-A 3′UTR, thus reduced tumor growth and suppressed tumor vascularization in a xenograft human gastric cancer model. The current findings suggest that miR-126 plays a vital role in regulating gastric cancer angiogenesis.

## RESULTS

### Changes of miRNA-126 expression and its relationship with MVD in gastric cancer tissue

**Figure 1:**
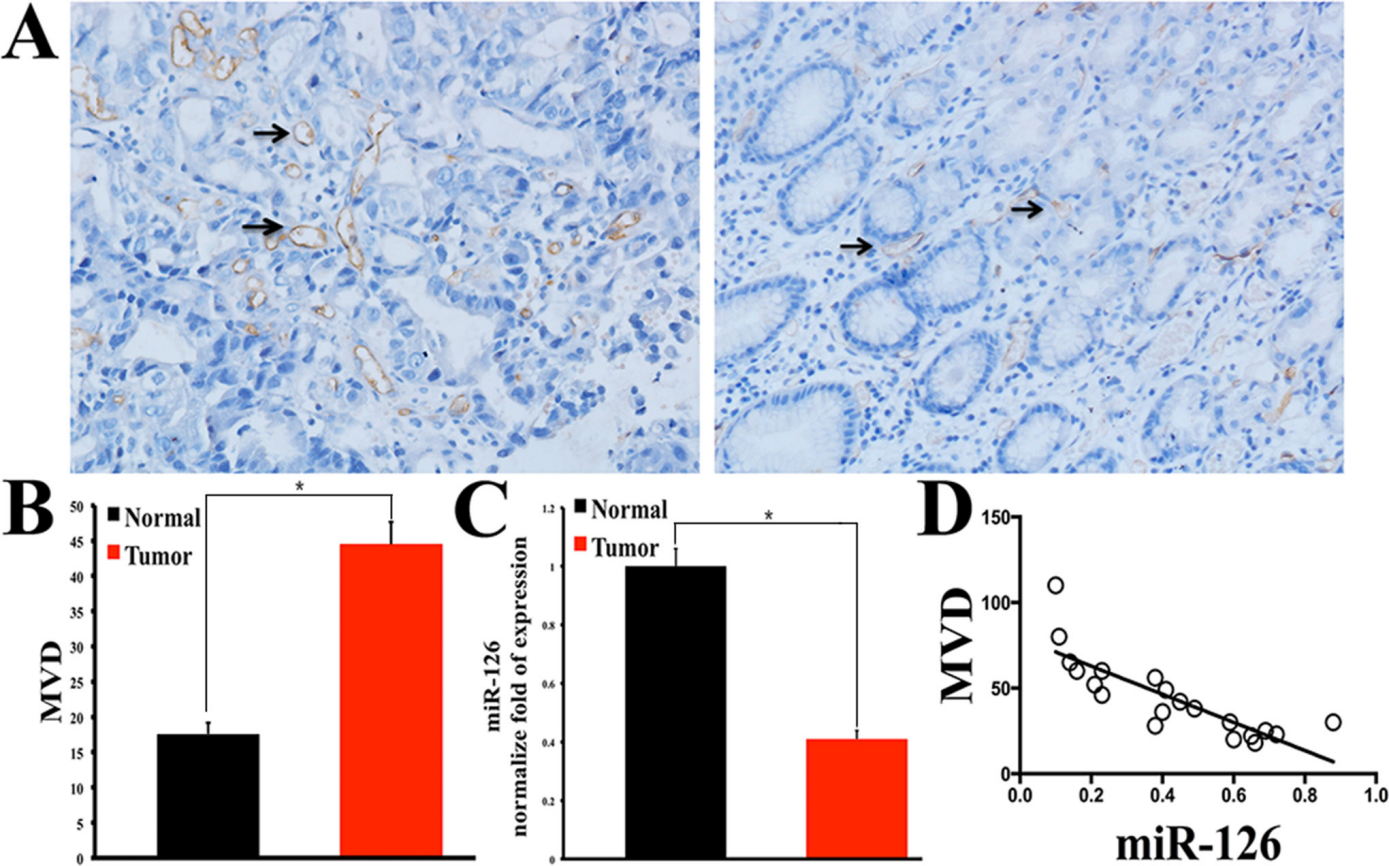
Microvessel density (MVD) is higher in gastric carcinoma tissue and is inversely correlated with miR-126 **(A)** Immunohistochemical staining of CD34 showing the expression of CD 34 is higher in human gastric carcinoma tissue than that in normal control gastric tissue (Left: gastric carcinoma tissue; Right: gastric normal tissue). **(B)** Bar graph summarizes the MVD, showing the MVD is higher in gastric carcinoma tissue than that in normal tissue (n = 68, *, *p* < 0.001). **(C)** Quantitative real-time RT-PCR results showing the miR-126 is lower in gastric carcinoma tissue than that in normal control tissue (n = 20, *, *p* < 0.01). **(D)** The correlation line showing that the MVD is inversely correlated with miR-126 (r = –0.8235, *p* < 0.001, n = 20).

To assess the neovascularization index, microvessel density (MVD) was determined by immunohistochemical staining of CD34 in 68 gastric adenocarcinoma tissues with matched normal gastric mucosas. As indicated in Figure 1A and B, the expression of CD 34 was much higher in human gastric carcinoma tissues than that in normal tissues. Furthermore, the expression levels of miR-126 from 20 fresh gastric carcinoma tissues and matched normal tissues were detected by the method of quantitative real-time reverse transcriptase-PCR assay (qRT-PCR). Compared with the normal tissues, the expression level of miR-126 was markedly reduced in all the 20 gastric carcinoma tissues (Fig. 1C). More importantly, the correlation line showed that the MVD was negatively correlated with miR-126 (Fig. 1D). These data strongly indicated that miR-126 may be involved in the angiogenic process of stomach cancer.

### miR-126 expression level reversely correlated with VEGF-A protein in gastric cancer

**Figure 2:**
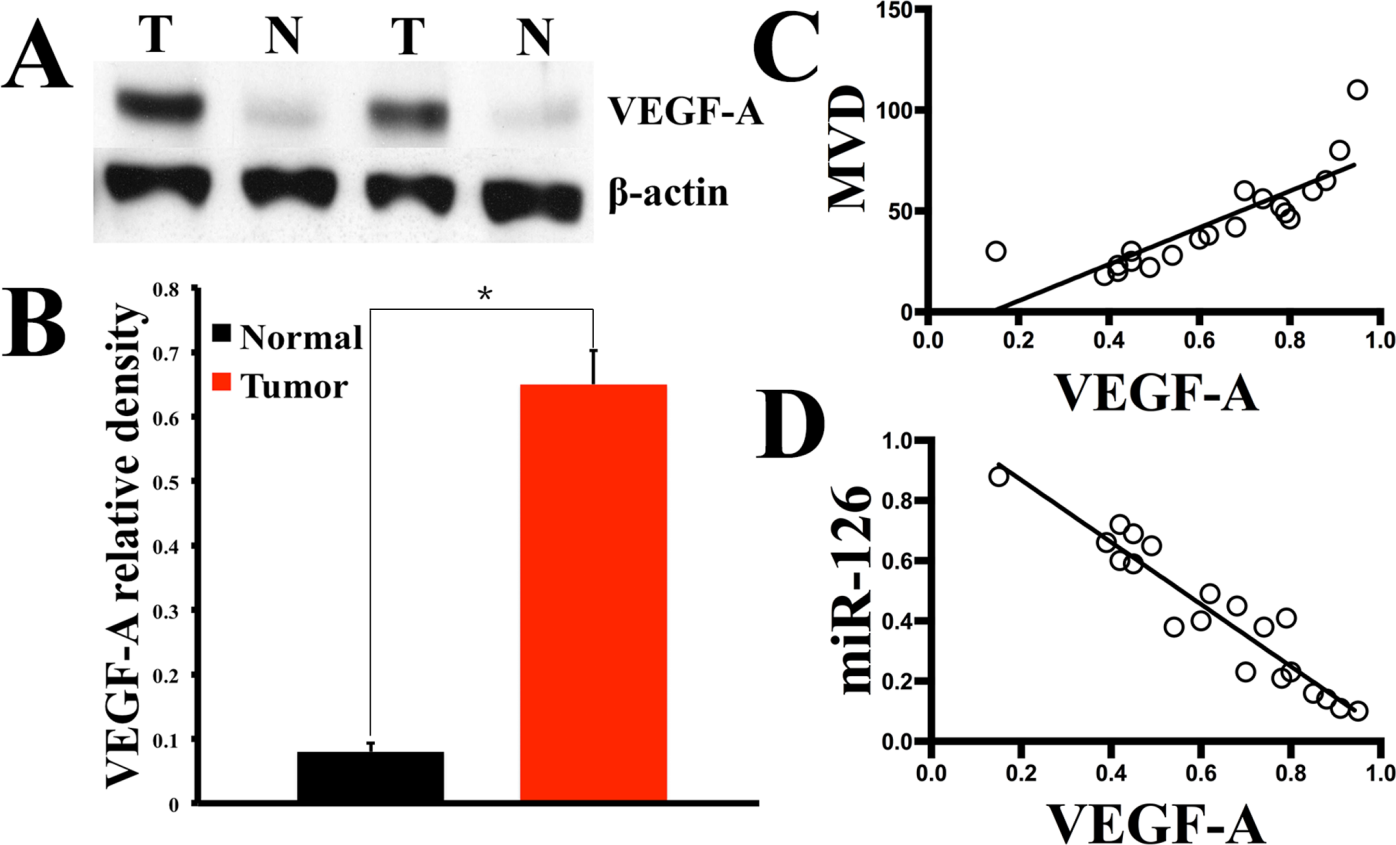
The expression of VEGF-A was enhanced in gastric cancer tissue and reversely correlated with miR-126 expression level Twenty gastric cancer tissues with matched normal gastric mucosas were acquired through surgical resection. Western blotting was used to test the relative expression level of VEGF-A **(A)**, the bar graph **(B)** shows that in all the 20 gastric carcinoma tissues, the mean expression level of VEGF-A is much higher than that of the normal tissues (n = 20, *, *p* < 0.001). Further analyzes revealed that the VEGF-A immunoblotting density was positively correlated with gastric cancer MVD index **(C)** detected by CD34 immunostaining (r = 0.8348, *p* < 0.0001, n = 20), while a inverse correlation was found between VEGF-A and miR-126 expression (**D**, r = –0.9480, *p* < 0.0001, n = 20).

To further clarify the role of miR-126 in the neovascularization of gastric cancer, we set out to determine whether miR-126 has relationship with angiogenic factors. It has been well documented that solid tumors cannot grow beyond a limited size without an adequate blood supply and VEGF plays a pivotal role in stimulating tumor new blood vessels formation. The most important VEGF family member is VEGF-A. Given the important role of VEGF-A in tumor angiogenesis, we conducted Western blot to analyze the VEGF-A expression in the above fresh 20 gastric carcinoma tissues and matched normal tissues. The Western-blot results showed higher expression of VEGF-A in gastric carcinoma tissues than normal ones (Fig. 2A and B). The relationships of VEGF-A to MVD and miR-126 expression level were further evaluated in gastric cancer. As results, VEGF-A was found to be positively correlated with MVD index (Fig. 2C) and negatively correlated with miR-126 (Fig. 2D) in gastric cancer tissue, which suggested a possible negative regulatory role of miR-126 in VEGF-A expression.

### miR-126 interacting with VEGF-A 3′UTR

**Figure 3:**
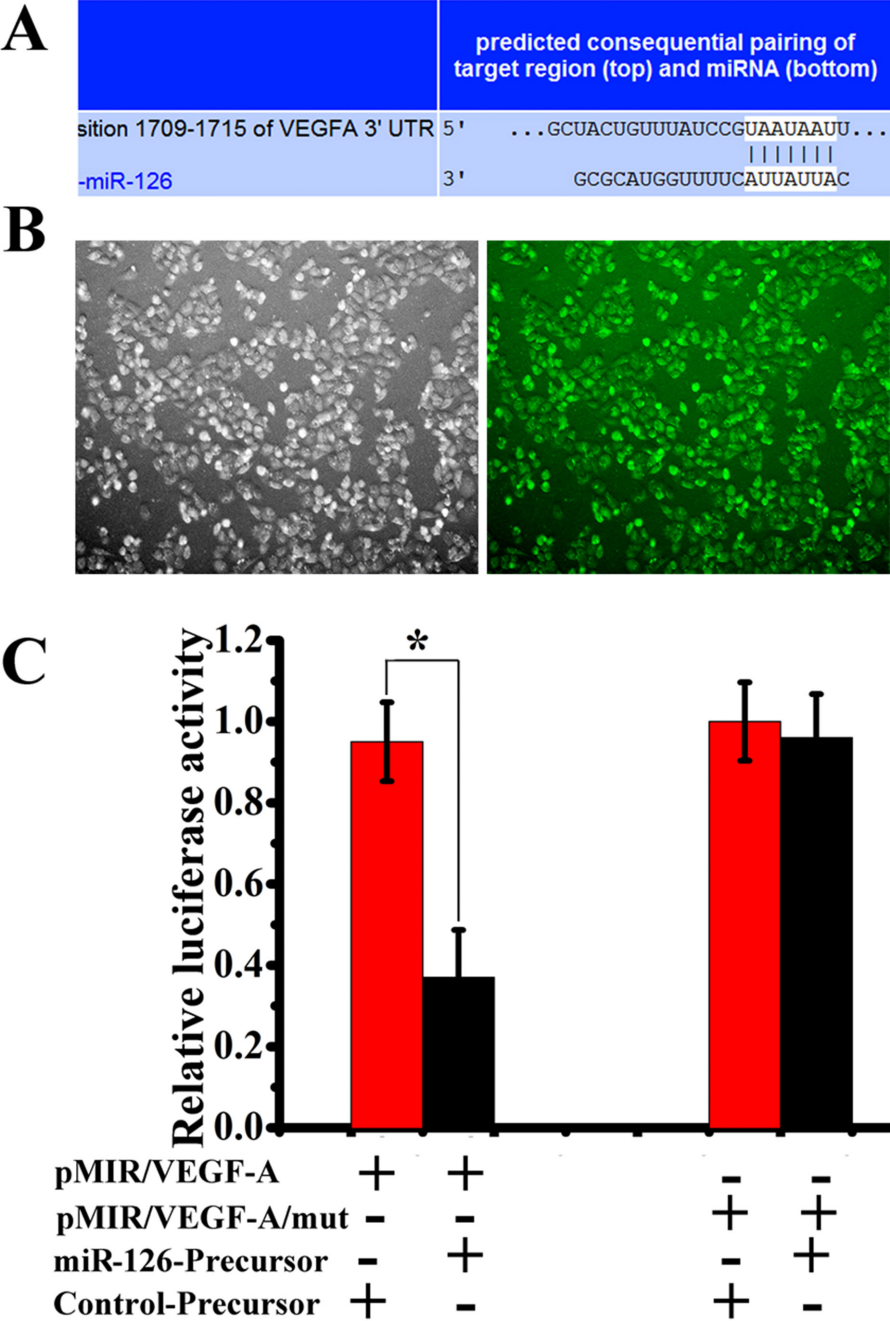
miR-126 interacts with VEGF-A **(A)** Putative binding sites of miR-126 in the VEGF-A 3′UTR predicted by TargetScan. **(B)**, Luciferase GFP positive in SGC-7901 cell lines. **(C)** miR-126 precursor down-regulated luciferase activities controlled by wild-type VEGF-A 3′UTR (n = 3, *, *p* < 0.01) but did not affect luciferase activities controlled by mutant VEGF-A 3′UTR (n = 3, *, *p* > 0.05). Data were obtained from 3 independent experiments.

To identify direct targets of miR-126, the two most-used public bioinformatic algorithms, TargetScan and miRanda, were used in combination. As shown in Figure 3A, VEGF-A is theoretically a potential target gene of miR-126, and the predicted binding site between miR-126 and VEGF-A 3′-UTR is also illustrated. In order to further test whether miR-126 is capable of regulating VEGF-A protein expression via the binding site in VEGF-A 3′UTR, we cloned the predicted miR-126 binding site from cDNA library downstream the firefly luciferase coding region in pMIR-REPORTTM Luciferase vector (pLuc, Ambion). Mutant of the putative binding site was also prepared. Another plasmid pLV-miR-126 was designed to deliver miR-126 contained a 655 bp genomic insert under control of the CMV promoter. SGC-7901 cells were cultured and transfected with pMIR/VEGF-A or pMIR/VEGF-A/mut with or without pLV-miR126-Precursor and control-Precusor. Forty-eight hours after transfection, cells were harvested and the protein was extracted for luciferase assay (Fig. 3B). The expression of miR126-Precusor via pMIR/VEGF-A/mut transfection reduced the firefly luciferase activity significantly (Fig. 3C). These results indicated that miR-126 could regulate VEGF-A protein expression via the binding site in VEGF-A 3′UTR.

### Inhibition of the VEGF-A expression and its downstream molecules in various gastric cancer cell lines via restoration of miR-126 expression

**Figure 4:**
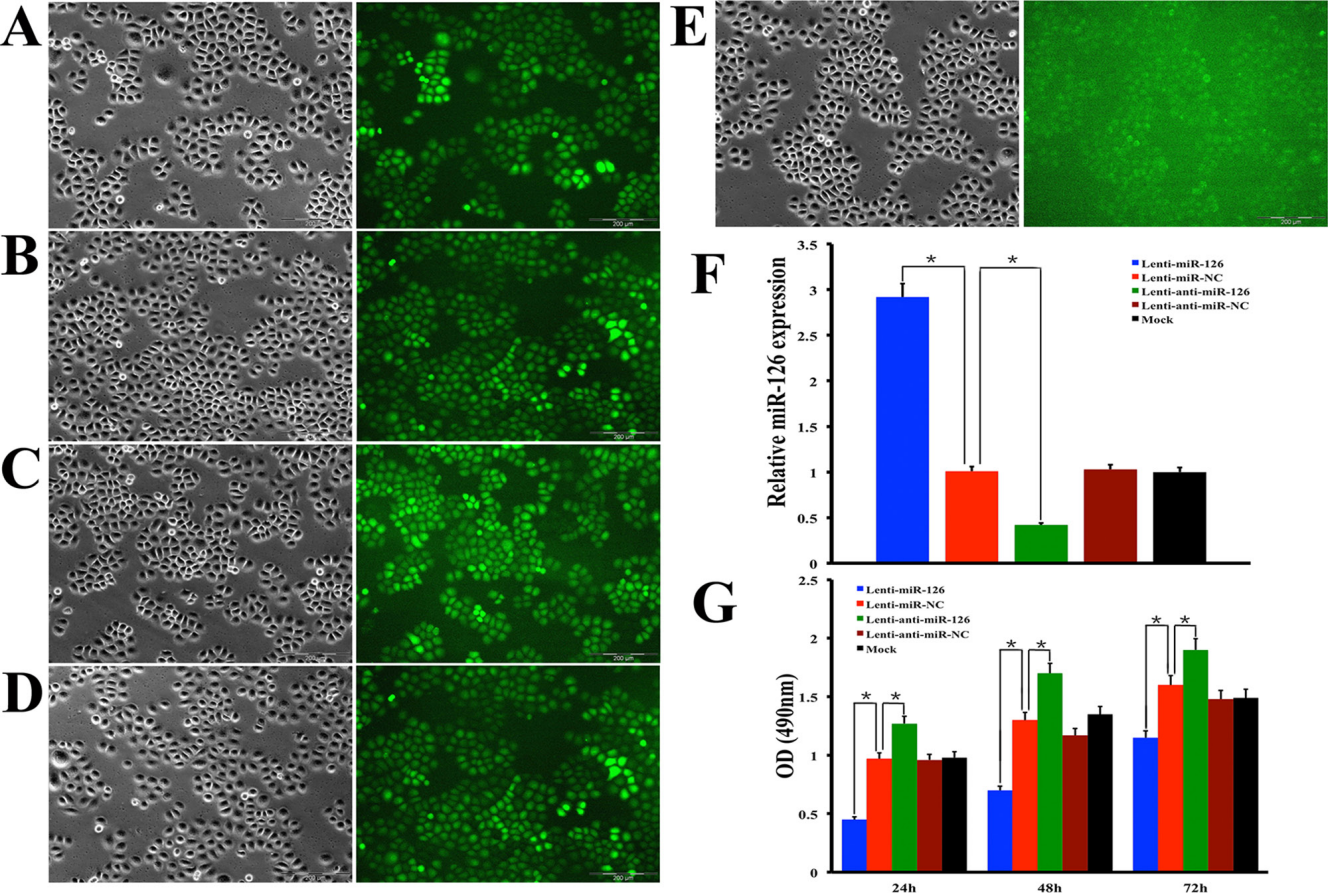
Lentivirus transfection of miR-126 increases the expression of miR-126 and inhibits gastric cancer cell proliferation SGC-7901 gastric cancer cells were stably transfected with Lenti-miR-126 **(A)** Lenti-miR-NC **(B)** lenti-anti-miR-126 **(C)** Lenti-anti-miR-NC **(D)** or treated only with enhanced infection solution (ENI.S) which served as mock control **(E)** respectively. Transfected cells were examined by phase contrast microscopy (left panel) and fluorescent microscopy (right panel) and high transduction efficiency was seen in these cells under fluorescent microscopy except the empty control group which no GFP positive cell was found. Bar graph **(F)** showing quantitative real-time RT-PCR confirmed the expression of miR-126 was dramatically decreased after Lenti-anti-miR-126 transfection and restored after Lenti-miR-126 transfection. The error bars are SD over 5 independent experiments (n = 5, *, *p* < 0.001). A MTT assay showed that Lenti-miR-126 suppressed cell proliferation, whereas Lenti-anti-miR-126 increases cell proliferation, respectively at 24h, 48h and 72h after transfection **(G)**. The error bars are SD over 5 independent experiments (n = 5, *, *p* < 0.01).

**Figure 5:**
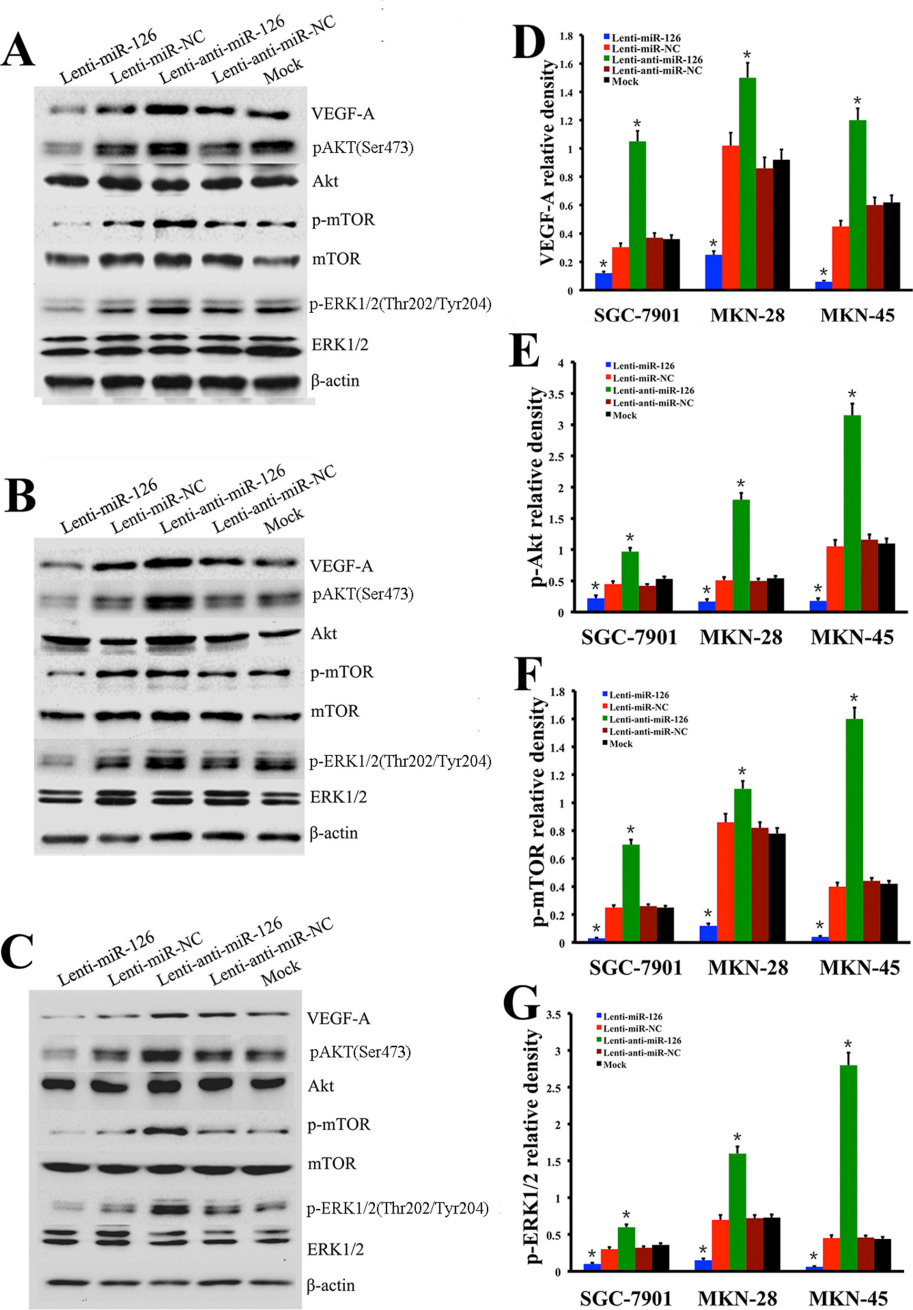
miR-126 expression affected VEGF-A and its downstream MAPK/ERK and Akt/m-TOR signaling pathway in gastric cancer cell lines Western blot analysis was performed to determine the expression of VEGF-A and its down stream proteins after restoration or inhibition of miR-126 expression by lenti-miR-126 or lenti-anti-miR-126 transfection in SGC-7901 **(A)**, MKN-28 **(B)** and MKN-45 **(C)** cells. Bar graph showing that, compared with the control groups, VEGF-A (D) and its downstream proteins p-Akt **(E)**, p-mTOR **(F)** and p-ERK1/2 **(G)** expression levels increased after inhibiting miR-126 expression, while the expression levels decreased after restoration of miR-126 in the above there gastric cancer cell lines. The data are representative of at least three different experiments. The error bars are SD over 5 independent experiments (n = 5, *, *p* < 0.01).

In order to confirm the regulatory role of miR-126 on VEGF-A expression in gastric cancer *in vitro*, SGC-7901 cells were infected by recombinant lentivirus miR-126 (Lenti-miR-126), lentivirus anti-miR-126 (Lenti-anti-miR-126) along with lentivirus miR negative control (Lenti-miR-NC), lentivirus anti-miR negative control (Lenti-anti-miR-NC) or transfection reagents without lentivirus vector (Mock), respectively (Fig. 4A-E). qRT–PCR was used to confirm the relative expression of miR-126 within these five cell groups. The results showed that the expression of miR-126 was decreased obviously in Lenti-miR-126 transfection cells while increased obviously in Lenti-anti-miR-126 transfection cells (Fig. 4F), but no obvious differences of miR-126 expression were observed among the three control group cells (Fig. 4F). MTT assay was employed to measure cell proliferation. The results shown in Fig. 4G suggested that exogenous expression of miR-126 could inhibit proliferation of SGC-7901 cells. Next, we detected the expression level of VEGF-A by the method of Western blot in SGC-7901 cells (Fig. 5A), the results showed that VEGF-A (Fig. 5D) and its downstream protein p-Akt (Fig. 5E), p-mTOR (Fig. 5F) and p-ERK1/2 (Fig. 5G) expression all increased after inhibiting miR-126 and decreased after restoration of miR-126 expression. To further clarify it, the experiments were also conducted in other two gastric cancer cell lines MKN-28 (Fig. 5B) and MKN-45 (Fig. 5C). Statistical analysis revealed a similarly effects of miR-126 on the expression of VEGF-A, p-Akt, p-mTOR and p-ERK1/2 to its effects on SGC-7901. These results suggested that miR-126 could inhibit VEGF-A expression *in vitro*.

### Effect of miR-126 expression on *in vivo* SGC-7901 tumorigenicity and angiogenesis

**Figure 6:**
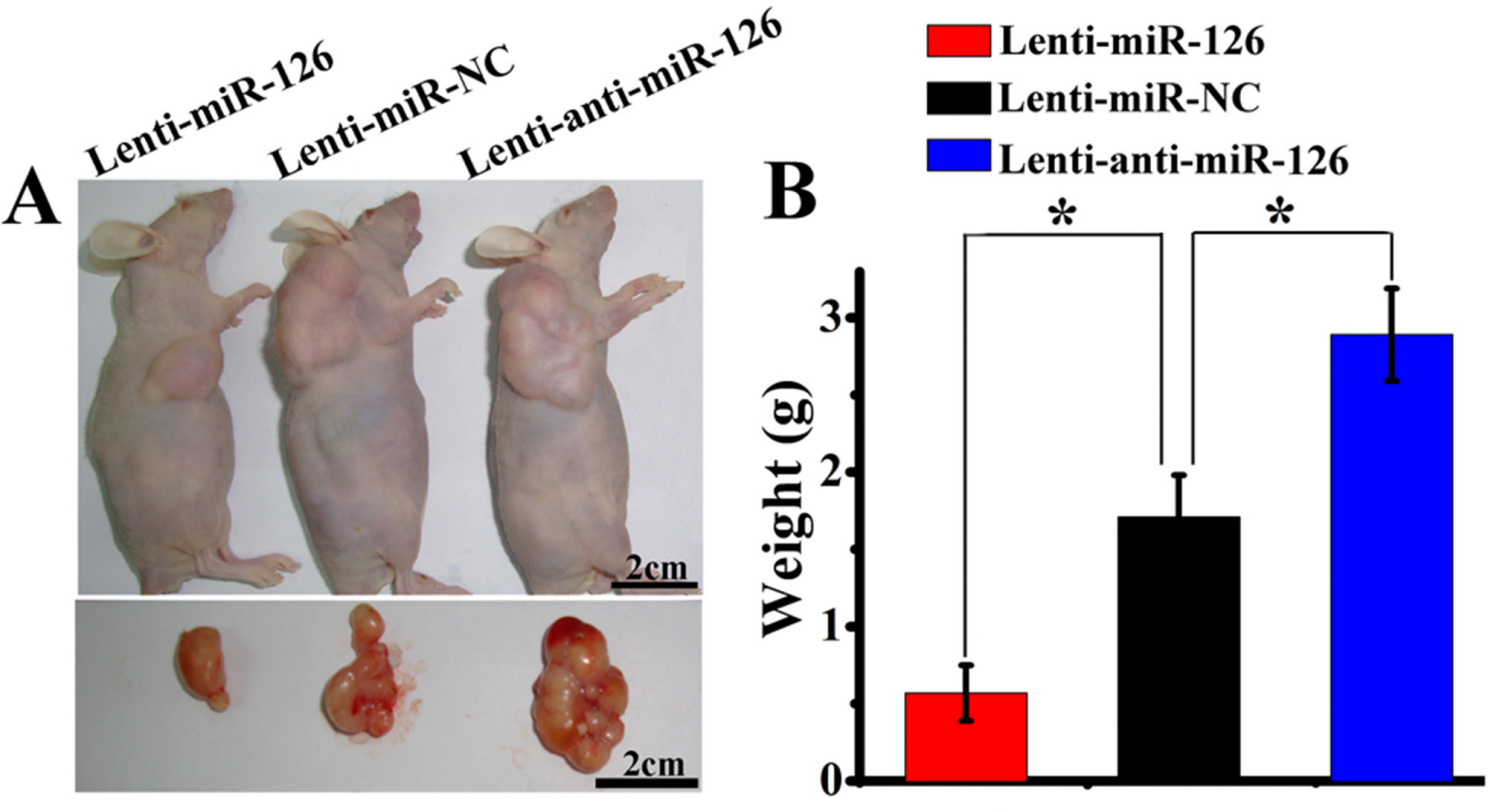
miR-126 expression affected tumorigenicity of gastric cancer cells SGC-7901 cells stably transfected with lenti-miR-126, lenti-miR-NC or lenti-anti-miR-126 were subcutaneously xenografted into three groups of nude mice. Representative images of xenografted mice bearing tumors and dissected tumors are shown **(A)**. The average tumor weight of miR-126 restoration group was lower than that in control group, while the weight in miR-126 down-regulated group was higher than that in control group (**B**, n = 8, *, *p* < 0.01).

**Figure 7:**
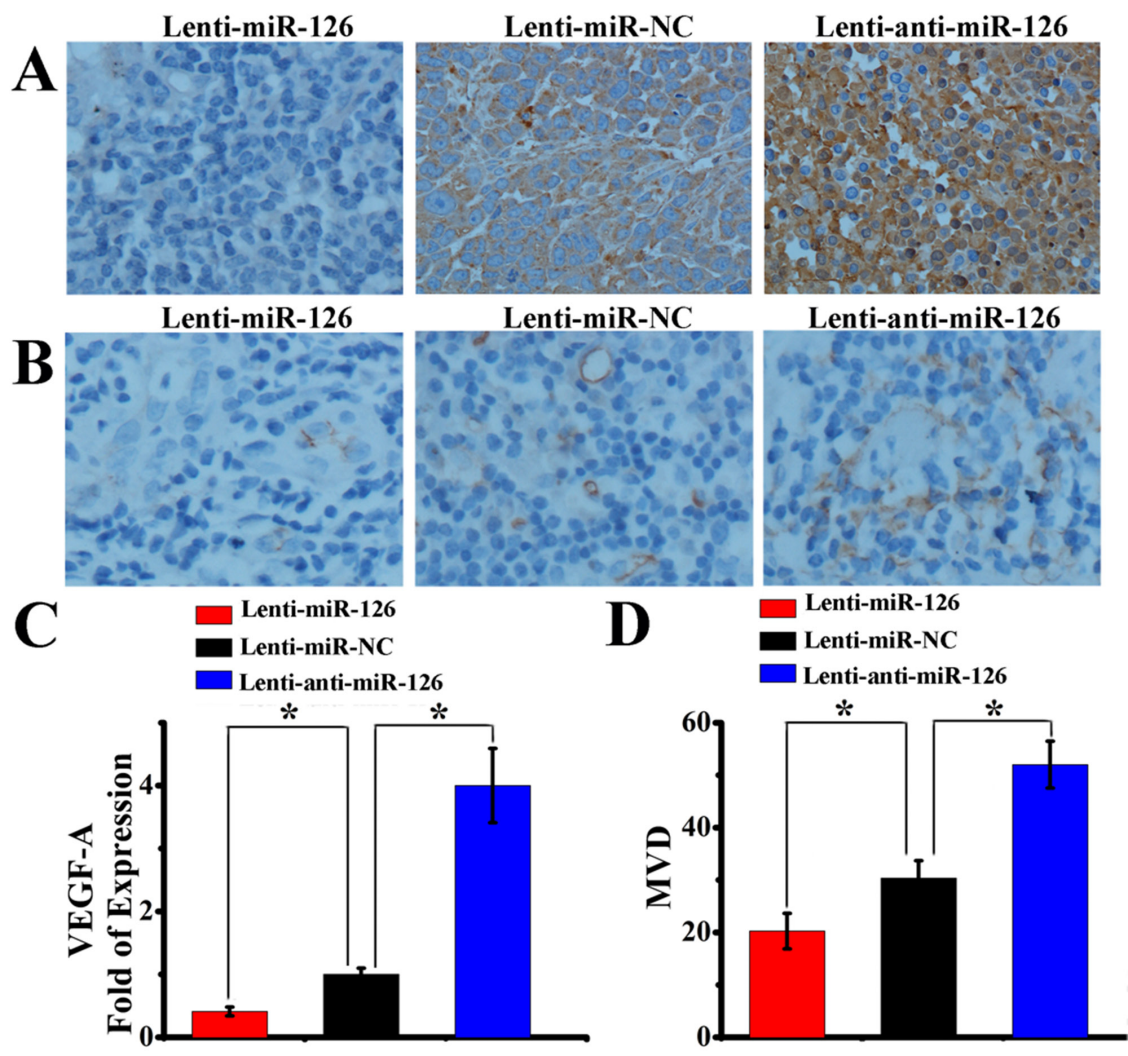
miR-126 restoration inhibited gastric cancer angiogenesis Immunohistochemical staining showed the *in situ* expression of VEGF-A **(A)** and CD34 **(B)** in gastric cancer mouse xenograft formed by three nude mouse groups inoculated with SGC-7901 cells stably transfected with Lenti-miR-126, lenti-miR-NC or Lenti-anti-miR-126. Semi-quantitative Western blot analysis revealed that the expression of VEGF-A increased after miR-126 interfered, while decreased after miR-126 up-regulated (**C**, n = 8, *, *p* < 0.01). The tumor MVD, which was quantified by CD34 expression, presented the same trend with VEGF-A after affecting miR-126 expression (**D**, n = 8, *, *p* < 0.05).

Angiogenesis is very important for the tumor growth. Next, we further tested whether changes of miR-126 expression could influence the growth of tumor *in vivo*. Three groups of nude mice were inoculated with SGC-7901 cells stably transfected with Lenti-miR-126, Lenti-miR-NC or Lenti-anti-miR-126. Tumor formation was observed and tumor weight was measured in these three groups. As a result (Fig. 6), ectopic expression of miR-126 inhibited tumorigenesis *in vivo*: the average tumor weight of mice inoculated with lenti-miR-126 transfected SGC-7901 cells at day 42 was 0.57 ± 0.21g, which was significantly lower (*P* < 0.05) than that of mice inoculated with lenti-anti-miR-126 transfected SGC-7901 cells (2.79 ± 0.31g) and lenti-miR-NC negative control group (1.73 ± 0.34g). Then, immunohistochemical detection of VEGF-A and CD34 expression in mice tumor xenograft was performed. As indicated in Figure 7A and C, the amount of VEGF-A antigen-positive cells was significantly lower in the tumor derived from lenti-miR-126 group than that in the control group and lenti-anti-miR-126 group. Accordingly, the amount of MVD determined by CD34 immunostaining (Fig. 7B and D) was lower in the tumor derived from miR-126 restoration group (20.05 ± 3.39) than that from the control group (30.35 ± 3.34) and miR-126 down-regulated group (52.00 ± 4.47). These results revealed that miR-126 might inhibit gastric cancer growth and angiogenesis by down-regulating VEGF-A expression.

## DISCUSSION

It has been demonstrated that abnormal expression of miR-126 is correlated with human tumorigenesis. Despite that previous studies found the suppressing role of miR-126 on gastric cancer growth, the potential role of miR-126 in gastric cancer angiogenesis remains unknown. In this study, we mainly investigated the role of miR-126 on gastric cancer angiogenesis. The results have shown the reduced expression of miR-126 associated with higher MVD and VEGF-A in gastric cancer. Furthermore, we found that miR-126 could interact with VEGF-A via the binding site in 3′UTR, and restoration of miR-126 expression in gastric cancer cell lines could not only reduce the VEGF-A expression *in vitro*, but also inhibit the tumor growth through decreasing the microvessel formation *in vivo*. Therefore, the present data from our studies indicate that miR-126 is a brake for gastric cancer angiogenesis, and dysfunction of miR-126 leads to tumor growth.

miR-126 was firstly found to be located in chromosome 9q34.3 within intron 7 for epidermal growth factor like-7 (EGFL-7) [32]. It is mainly expressed within the vascular endothelial cells, and functions in tuning angiogenic process and vascular formation during normal development and injury healing [21, 33, 34]. In normal vasculogenesis, up-regulated miR-126 in endothelial cells could enhance VEGF activity and promote vessel formation by repressing the expression of sprouty-related protein-1 (Spred-1), and knockdown of miR-126 leads to destruction of vascular integrity and hemorrhage during embryonic development [21, 22]. In contrast, it has been found that down-regulated miR-126 increases VEGF-A activity in lung cancer, oral cancer and breast cancer [30, 35–37], and restoration of miR-126 can decrease VEGF and tumor size in lung cancer [30, 31]. For gastric cancer, miR-126 was also found to associated with clinic pathological features, including tumor size, lymph node metastasis, local invasion and tumor-node-metastasis (TNM) stage. However, there were some controversies for miR-126 in gastric cancer. miR-126 was identified as a tumor suppressor in one study [14], while the results from another study found miR-126 contributed to gastric carcinogenesis [38]. These contradictory results suggest that miR-126 might be expressed in a tissue-specific pattern and have cell content-dependent functions. Anyway, the controversial findings concerning miR-126 confirm its important role in tumourigenesis and progression, regardless of its precise tumourigenic or tumour suppressive nature.

In this study, we further investigated the role of miR-126 in gastric cancer. We found that miR-126 was down-regulated in gastric cancer samples compared with normal samples, as indicated by qRT-PCR analysis, consistent with the previous analysis [14]. Previous studies demonstrated that increased VEGF-A level was related to worse prognosis in most aggressive solid tumors including gastric cancer [39, 40], and it was closely associated with tumor progression and angiogenesis [41, 42]. Our further analysis in gastric cancer tissues revealed that the expression of miR-126 was reversely correlated with MVD and VEGF-A protein expression. Thus, miR-126 may function as a tumor suppressor through regulation of VEGF-A in gastric cancer. miRNAs usually directly inhibit the mRNA of their target genes by competitively binding with 3′UTR site in targeted mRNA [43]. To further investigate whether VEGF-A is a target gene of miR-126, we searched for miR-126 target genes using bioinformatic analysis and found VEGF-A had a putative miR-126 binding site within its 3′UTR. We identified VEGF-A as an miR-126 target gene in gastric cancer cells based on the results of the luciferase reporter assay. Recently, several novel targets of miR-126 have been confirmed including PI3KR2 [44], Crk [44], IκBα [45], IRS-1 [46], ADAM9 [47], CXCR4 [26], Spred-1 [48] and SOX2 [38]. Notably, VEGF-A has also been reported to be a target gene of miR-126 in human breast cancer [36], non-small cell lung cancer [35], oral cancer [37], colorectal cancer [49] and hepatic stellate cells [50]. Here, we reported that VEGF-A was also a target gene of miR-126 in human gastric cancer. To further clarify it, we detected the VEGF-A expression level in three gastric cancer cell lines, which transfected with lentivirus vectors to up- or down-regulate miR-126 level. As a result, the expression of VEGF-A and its downstream signaling molecules (p-Akt, p-mTOR and p-ERK) as well as cell proliferation reversely correlated with miR-126 expression. Akt and ERK are well known kinases that activate and promote cell proliferation by stimulating growth factors. Previous studies revealed that VEGF promotes angiogenesis through the activation of MAPK/ERK and Akt/m-TOR signaling pathway in ovarian cancer, hepatocellular carcinoma and non-small-cell lung cancer [51–53]. Now the results from our *in vitro* experiment also suggested that MAPK/ERK and Akt/m-TOR signaling pathway involed in miR-126/VEGF-A signaling pathway in gastric cancer.

Lots of evidences showed that VEGF significantly promoted the division, proliferation, and migration of endothelial cells [54, 55]. It has become an important mediator for cancer growth and metastasis, and many new therapies were designed to rectify VEGF activity [56]. As accumulation of knowledge on miRNAs in carcinogenesis, these molecules might be used in clinical diagnostic and prognosis prediction [10, 57]. As the important role of miR-126 we found on VEGF-A in gastric cancer, we further validated the *in vitro* data by using a xenograft mouse model, showing that tumor growth as well as VEGF-A and MVD was significantly suppressed in Lenti-miR-126 stablely transfected SGC-7901 xenografts as compared with control group. However, the regulation of miR-126 expression is totally unknown in gastric cancer. More detailed function of miR-126 in oncogenesis of gastric cancer should be further investigated in future studies.

In conclusion, reduction of miR-126 played an important role in angiogenesis of gastric cancer, and ectopic expression of miR-126 could significantly inhibit VEGF-A expression, thus counteract the proliferation and growth of gastric cancer cells both *in vitro* and *in vivo*. Understanding how miR-126 is involved with gastric cancer pathogenesis will be useful in developing potential therapeutic targets in the management of gastric cancer.

## METHODS

### Cell culture and tissue collection

The human GC cell lines SGC-7901, MKN-28 and MKN-45 were provided by Digestive Disease Institute of Nanchang University. It was grown in RPMI 1640(Gibco, Grand lsland, NY) supplemented with 10% fetal bovine serum (Gibco, Grand Island, NY). 68 consecutive patients undergoing surgical resection for gastric adenocarcinoma at the First Affiliated Hospital, Nanchang University, Nanchang, China, between August 2011 and January 2012, were recruited to the study. Patients who had received any chemo-, radio- or immunotherapy before surgery were excluded from the study. Samples of gastric adenocarcinoma and matched normal gastric mucosa (≥10 cm from the distal tumor margin) were collected from each patient and flash-frozen in liquid nitrogen. A portion of each sample was fixed in 10% buffered formalin, embedded in paraffin wax, and cut into 3-μm thick sections for histopathological analysis (hematoxylin and eosin staining) and immunohistochemical staining. The remnant flesh samples were used for Western blotting and qRT-PCR. All methods are described below. The study was carried out in accordance with the Declaration of Helsinki (2000) and was approved by the Ethics Committee of Nanchang University. Written informed consent was obtained from all participants.

### Generation of stable transformants

Lentiviral constructs containing miR-126 (Lenti-miR-126) and anti-miR-126 (Lenti-anti-miR-126) along with miR negative control (Lenti-miR-NC) and anti-miR negative control (Lenti-anti-miR-NC) were designed and provided by Genechem lnc.(Shanghai, China). 70–80% confluent cells were transfected with lentivirus at multiplicity of infection (MOI) of 20 with enhanced infection solution (ENI.S) according to the manufacturer's protocol. Stably transfected cells were selected with 1ug/ml puromycin (Sigma, German). Stable transformants were identified by fluorescence microscopy and qRT-PCR.

### MTT assay

Cell proliferation was analysed using a 3-(4,5-dimethylthiazol-2-yl)-2,5-dipheny ltetrazoliumbromide (MTT) assay. Cells were seeded into 96-well plates (5 × 10^3^ cells/well) directly or at 24 h after stable transfection and incubated for 24h, 48h and 72h, respectively. After incubation with 25 μl of MTT (5 mg/ml, Sigma, USA) at 37°C for 4 h, the supernatants were removed, and 150 μl of dimethylsulfoxide (DMSO, Sigma, USA) was added to each well. The absorbance value (OD) of each well was measured at 490 nm. For each experimental condition, 6 wells were used, and the experiment was performed in 5 times.

### Luciferase reporter assay

The pMIR-REPORTTM Luciferase vector (pLuc, Ambion) were used for reporter assay. Cells were cultured and transfected with pMIR/VEGF-A or pMIR/VEGF-A/mut with or without pLV-miR126-Precursor and control-Precusor plasmids using Lipofectamine reagent (Invitrogen). All transfections were performed in triplicate. After forty-eight hours transfection, the medium was aspirated and cells were lysed with a mixture of 15 μL Luciferase Assay Reagent II (Promega) and 15 μL nuclease-free water (Invitrogen). Firefly luciferase activity was measured after 10 min. Then, 15 μL Stop & Glo Reagent (Promega) were added and *Renilla* luciferase activity was measured after 10 min. Luciferase activity measurements were performed in an LMAX II 384 luminometer (Molecular Devices), with 5 seconds integration time. For each triplicate, the mean *Renilla*/firefly ratio was calculated.

### Quantitative real-time reverse transcriptase–PCR assay

Total RNA from tissue samples or cells was extracted by using Trizol reagent (Invitrogen, CA). RNA was first reversely transcribed into cDNA by using RT reagent Kit (TOYOBO, Japan). Then the cDNA was subjected to RT-PCR with an SYBR Green RT-PCR Master Mix kit (TOYOBO, Japan) in an ABI PRISM 7500 system (Applied Biosystems, USA) by using the miR-126 primers set and U6 primers set (Ribobio, China). The primer sequence of miR-126 is 5′- CATTATTACTTTTGGTACGCGAAA-3′. The relative levels of miR-126 transcripts were normalized to the control U6 mRNA, and the primer sequence was 5′-TCGTGAAGCGTTCCATATTTTTAA-3′. All samples were normalized to internal controls, and the relative expression level was calculated through the 2^−ΔΔCt^ analysis method. Experiments were performed in triplicate samples. Relative gene expression was quantified using the GraphPad Prism 4.0 software (GraphPad Software, San Diego, CA, USA) and expressed as a percentage of the control.

### Western blot

For Western blot analysis, the cells in culture were lysed using the RIPA buffer (Pierce, Rockford, IL, USA) in the presence of Protease Inhibitor Cocktail (Pierce). Tissue samples were lysed using the T-PER Tissue Protein Extraction Reagent (Pierce) in the presence of Protease Inhibitor Cocktail (Pierce). The protein concentration of the lysates was measured using a BCA Protein Assay Kit (Pierce). Equivalent amounts of protein were resolved and mixed with 5X Lane Marker Reducing Sample Buffer (Pierce), electrophoresed in a 10% SDS–acrylamide gel and transferred onto nitrocellulose membranes (Santa Cruz Biotechnology, USA). The membranes were blocked with 5% non-fat milk in tris-buffered saline. Then the membranes were first probed by VEGF-A monoclonal antibody (Abcam, USA) and subsequently with HRP-conjugated secondary antibodies. Protein bands were visualized using enhanced chemiluminescence (ECL Western blotting detection, Amersham Life Science, Amersham, UK) and exposure to medical X-radiographic film for 1 min. Films were scanned (Epson GT-9500, Japan) and protein bands were quantitated by densitometry (Image PC alpha 9; National Institutes of Health, Bethesda, MD, USA) with reference to β-actin levels. VEGF downstream modulates signaling, including intracellular signaling Akt (Santa Cruz Biotechnology), mammalian target of rapamycin (mTOR) (Abcam, USA), and extracelluar signal-regulated kinase 1/2(ERK1/2) (Santa Cruz Biotechnology, USA) were also investigated according to the protocol.

### *In vivo* tumor xenograft model

To confirm miR-126 function *in vivo*, 1 × 10^7^ SGC-7901 cells stably transfected with lenti-miR-126, lenti-miR-NC or lenti-anti-miR-126 were injected subcutaneously into the right armpit of three groups of 18–26g male BALB/c nude mice (8 mice/group). At 42 days after inoculation, all mice were sacrificed. The tumor masses were excised, weighed, photographed and subjected to immunohistochemistry and Western blot, using CD34 antibody (Maxim Biotech, USA) and VEGF-A antibody (Abcam, USA). All animal experiments were performed in accordance with institutional guidelines and were approved by the animal care review board at the Nanchang University.

### Quantification of microvessel density

Tissue sections were de-paraffinized, rehydrated and incubated in 3% hydrogen peroxide for 15 min to quench endogenous peroxidase. Slides were heated in a microwave oven for 10 min in 10 mM citrate buffer (pH 6.0), then incubated with rabbit monoclonal antihuman CD34 (1 : 40 dilution; Maxim Biotech, San Francisco, CA, USA) overnight at 4°C. Slides were washed three times with 0.01 M PBS (pH 7.35) for 3 min each wash, incubated with peroxidase- labelled goat antirabbit IgG (1 : 200 dilution, DakoCytomation) for 10 min at 37°C and washed three times with PBS (3 min each wash). Immunostaining was visualized with DAB and sections were counterstained with hematoxylin. Tumor microvessel density was determined as described [58]. In brief, the area of most intense neovascularization (preferably on the tumor margin) was selected by scanning on low magnification (× 10–100). Only the vascularity of tumor areas considered to be viable (i.e. non- necrotic) was taken into account. Individual microvessel was counted at × 200 magnification (0.723 mm2/field). Any brown-stained, nucleus-containing endothelial cell that was clearly separate from adjacent microvessel, tumor cells and other connective tissue elements was considered a single, countable microvessel, without requirement for a lumen or the presence of erythrocytes. Each patient's microvessel count was the mean of two separate counts made by two different pathologists who remained blind to patient outcome.

### Statistical analysis

Results were analyzed statistically using Student's *t*-test for comparisons between two groups. Data are presented as the means ± SDs. Correlation parameters were submitted to Pearson and non-parametric Spearman correlations. A *P* value less than 0.05 was considered to indicate statistical significance.
